# Coevolution in a One Predator–Two Prey System

**DOI:** 10.1371/journal.pone.0013887

**Published:** 2010-11-09

**Authors:** Akihiko Mougi

**Affiliations:** Department of Biology, Faculty of Sciences, Kyushu University, Fukuoka, Japan; University of Bristol, United Kingdom

## Abstract

**Background:**

Our understanding of coevolution in a predator–prey system is based mostly on pair-wise interactions.

**Methodology and Principal Findings:**

Here I analyze a one-predator–two-prey system in which the predator's attack ability and the defense abilities of the prey all evolve. The coevolutionary consequences can differ dramatically depending on the initial trait value and the timing of the alternative prey's invasion into the original system. If the invading prey species has relatively low defense ability when it invades, its defense is likely to evolve to a lower level, stabilizing the population dynamics. In contrast, if when it invades its defense ability is close to that of the resident prey, its defense can evolve to a higher level and that of the resident prey may suddenly cease to evolve, destabilizing the population dynamics. Destabilization due to invasion is likely when the invading prey is adaptively superior (evolution of its defense is less constrained and fast), and it can also occur in a broad condition even when the invading prey is adaptively inferior. In addition, invasion into a resident system far from equilibrium characterized by population oscillations is likely to cause further destabilization.

**Conclusions and Significance:**

An invading prey species is thus likely to destabilize a resident community.

## Introduction

Coevolution, reciprocal phenotypic changes in interacting species over generations, is believed to be a major source of species diversity [1], [2]. Antagonistic interactions such as predator–prey interactions are one type of selective force causing coevolution [3]. A strong relationship between species, such as that between a specialist predator and its prey, can cause their interaction traits to change continuously, a phenomenon known as Red Queen dynamics [4]–[9]. Such tight, pair-wise coevolution is one extreme; the other is called diffuse coevolution [10].

Coevolution must be studied in simplified systems, because in general few species in a community have strong relationships that exert strong selection pressure [11]–[16], and also from the point of view of testability [17]. However, our understanding of coevolution is based almost entirely on pair-wise interactions [18]–[19]. Few theoretical studies have examined the impact of a third species with a fixed trait on the focal pair-wise coevolutionary dynamics [20]–[22], or on coevolution in three species [23]–[26]. In particular, there are few studies focusing on a question how the invasion of third species influences coevolutionary dynamics in the resident community.

Here I model a simple one-predator–two-prey system in which the attack ability of the predator and the defense abilities of the prey species evolve, and show that the coevolutionary consequences can be dramatically different depending on the initial trait value of the alternative prey and the timing of its invasion into the original predator–prey coevolutionary system. The invasion is likely to cause destabilization of the population dynamics over a wide range of trait values even when the invading prey is adaptively inferior (i.e., the evolution of its trait is more constrained and slower) to the resident prey species. In addition, if the invasion occurs when the resident predator–prey system is unstable, further destabilization is likely to occur. These results suggest that an invading species is likely to destabilize a resident community.

## Methods

### Population dynamics

I consider the following one-predator–two-prey system [27]–[29],
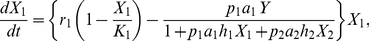
(1a)

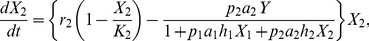
(1b)

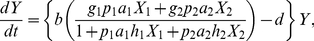
(1c)where *X_i_* (*i* = 1 or 2) and *Y* are prey and predator population sizes, respectively; *r_i_* is the per capita prey growth rate; *K_i_* is the carrying capacity of the prey; *a_i_* is the searching efficiency for prey *i*; *h_i_* is the handling time of prey *i*; *g_i_* is the energy value of an individual of prey *i*; *b* is the conversion efficiency, which relates the predator's birth rate to prey consumption; and *d* is the death rate of the predator. On encounter, predator decides to attack the prey with probability *p_i_*. This type 2 functional response has been used in the case where predator plays optimal foraging. In this study, I assume that any prey is attacked upon encounter (*p_i_* = 1) for the simplicity. I also assume that there is no interspecific competition between two preys for the analytical simplicity.

Parameters *r_i_*, *a_i_*, and *b* are functions of certain traits of the two species. I assume that *a_i_* is a function of the predator trait *v* and the prey trait *u_i_*: that is, *a_i_*(*u_i_*, *v*). *a_i_* decreases as the difference between *u_i_* and *v* increases, which is an appropriate model for trait interactions such as speed–speed, weapon–armor, and toxin–antitoxin [7]. Specifically, *a_i_* is the sigmoidal function, *a_i_* = *a*_0_/(1 + exp[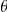
(*u_i_*–*v*)]), where *a*_0_ is the maximum capture rate and 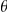 is the shape parameter of the function. As 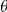 increases, the function approaches a step function. If the value of the prey's trait *u_i_* is much greater than that of the predator's trait *v*, the prey can escape predation effectively, and *a_i_* is very small. In contrast, if the value of the predator's trait *v* is much greater than that of the prey's trait *u_i_*, then the capture rate *a_i_* is large.

The cost of developing the trait in each species is modeled by assuming that *r_i_* and *b* are decreasing functions of *u* and *v*, respectively (trade-off functions). The rates of decrease (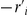 and 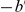) indicate the strength of the cost constraint on the prey and the predator, respectively. I adopted the non-linear functions *r_i_* = *r*_0_(1–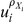) and *b* = *b*_0_(1–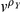), where *r*_0_ and *b*_0_ are the basal per capita prey growth rate and the basal conversion efficiency of the predator, respectively, and 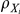 and 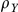 represent the strength of the trade-off in the prey and the predator, respectively.

In this paper, I assume *g*_1_ = *g*_2_ = 1 without loss of generality. I also assume *K*_1_ = *K*_2_ and *h*_1_ = *h*_2_ for the simplicity.

### Evolutionary dynamics

I model the evolutionary dynamics of the population mean trait values, *u_i_* and *v*, using a quantitative trait evolution model [28], [30] as follows:
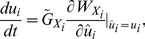
(2a)

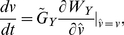
(2b)where 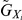 and 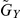 represent the speed of evolutionary adaptation, which is equal to the additive genetic variance divided by the generation time, in the prey and predator, respectively. In this model, the genetic variance is assumed to be always kept by the factors such as mutation and immigration. For simplicity, I assume the speed is constant (see the references [31], [32] for a discussion of the case that the genetic variances can change). *W_Xi_* and *W_Y_* are prey fitness and predator fitness, respectively, defined as the per capita rate of population growth:
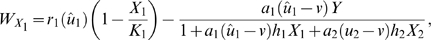
(3a)

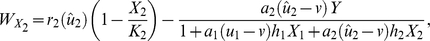


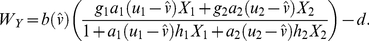
(3b)
*W_Xi_* is a function of trait 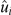 of the focal prey individual, and *W_Y_* is a function of trait 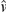. They also depend on the population mean traits *u_i_* and *v* [28], [30]. Equation (2) indicates that the rate of adaptive change in the traits should be proportional to the selection gradient. If the selection gradient is positive (negative), selection pushes the population toward higher (lower) trait values. At evolutionary equilibrium, Eq. (2) becomes zero.

The six differential equations, (1) and (2), describe the coupled coevolutionary and ecological dynamics of two prey and one predator species, which I analyze further below.

## Results

### Pair-wise coevolution

**Figure 1:**
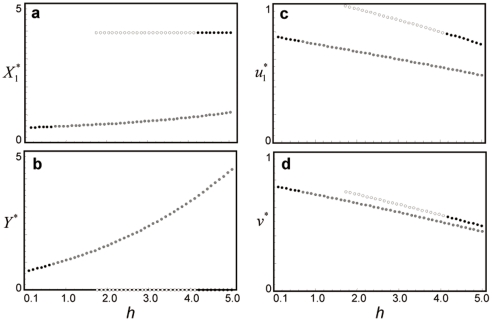
Equilibrium values in the one predator–one prey system, without prey species 2, in relation to handling time. The black, white, and gray points indicate locally stable and unstable equilibria and a limit cycle, respectively. Parameter values are *r*_0_ = 1, *K*_1_ = 4, *b*_0_ = 1, *g*_1_ = 1, *h*_1_ = 0.5, *a*_0_ = 1, 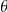 = 7.4, *d* = 0.1, 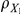 = 1.85, 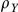 = 1.5, 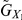 = 0.1, and 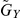 = 0.005.

**Figure 2:**
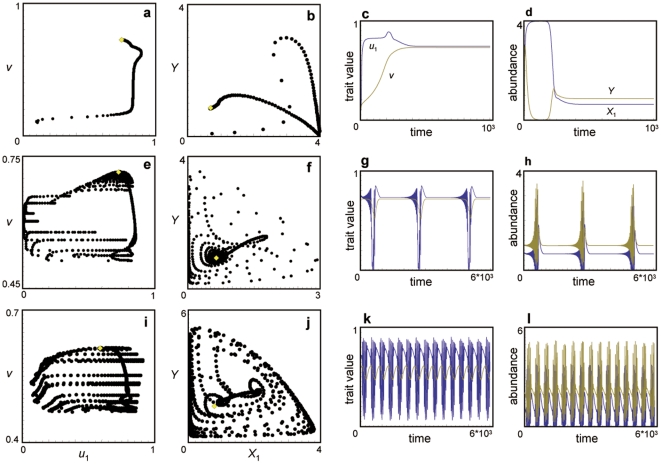
Phase plots and time series of the coevolutionary dynamics of the one predator–one prey system. (a–d) *h*_1_ = 0.5. (e–h) *h*_1_ = 0.7. (i–l) *h*_1_ = 2.3. The other parameter values are the same as in Fig. 1. The yellow diamond indicates the equilibrium point. The blue and yellow curves respectively show the population and trait dynamics of prey species 1 and the predator.

When an alternative prey (species 2) invades a community in which one predator and one prey (species 1) coexist, the original predator–prey pair may either coexist stably or their populations may oscillate (Fig. 1). When the handling time *h*_1_ is short, the population sizes (*X*_1_ and *Y*) and trait values (*u*_1_ and *v*) converge to an equilibrium (Fig. 2a–d). When *h*_1_ has an intermediate value, they alternate between a steady state and an oscillatory state (Fig. 2e–h). When *h*_1_ is long, they oscillate continuously (Fig. 2i–l). I consider the invasion of the second prey species into each of these qualitatively different systems.

### Three-species coevolution

**Figure 3:**
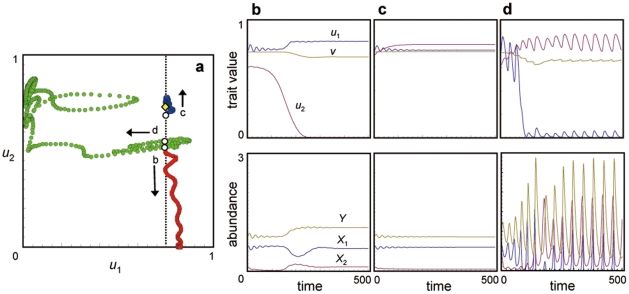
Three-species coevolutionary dynamics after the invasion of prey species 2 into a stable one predator–one prey system. (a) Phase plot of the coevolutionary dynamics. The yellow diamond indicates the equilibrium point in the absence of prey species 2, where it indicates the point where *u*_2_ equals the equilibrium 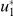. Also, the vertical dashed line indicates 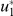; the white circles indicate the initial coordinates of *u*_2_; and b–d indicate the trajectories (each is shown by a different color and their directions are shown by arrows) of the coevolutionary dynamics from each initial point. In the trajectories b and c, the initial population size of *X*_2_ is 0.1, and in trajectory d, that is 0.5 (see also Fig. S1). (b–d) Time series of population sizes and trait values for trajectories b–d. The blue, red, and yellow curves respectively show the population and trait dynamics of prey species 1 and 2 and the predator. Parameter values are *r*_0_ = 1, *K*_1_ = *K*_2_ = 4, *b*_0_ = 1, *g*_1_ = *g*_2_ = 1, *h*_1_ = *h*_2_ = 0.5, *a*_0_ = 1, 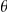 = 7.4, *d* = 0.1, 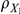 = 1.85, 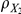 = 1.5, 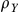 = 1.5, 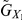 = 0.1, 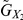 = 0.01, and 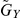 = 0.005.

First, a second prey with a small population size is introduced into an original one predator–one prey system in a globally stable equilibrium state (yellow diamond in Fig. 3a, S1a, b). If the initial defense trait value of the second prey is lower than the equilibrium trait value of the resident prey (see the initial point of trajectory b in Fig. 3a), the trait of the second, invading prey evolves to a lower value after it invades and three species coexist stably (trajectory b in Fig. 3a; Fig. 3b). If the initial trait value of the invading prey is close to the equilibrium trait value of the resident prey (see the initial point of trajectory c in Fig. 3a), the defense trait of the invading prey evolves to a higher value after it invades and three species coexist stably (trajectory c in Fig. 3a; Fig. 3c). These two patterns are the only ones observed when the initial population size of the second, invading prey is very low (Fig. S1d). When the initial population size of the second prey is relatively large (Fig. S1d), however, a new pattern emerges: the second prey evolves its defense to higher levels, and the resident prey, in contrast, evolves its defense to a much lower level; these changes result in destabilization of the population dynamics (trajectory d in Fig. 3a; Fig. 3d).

**Figure 4:**
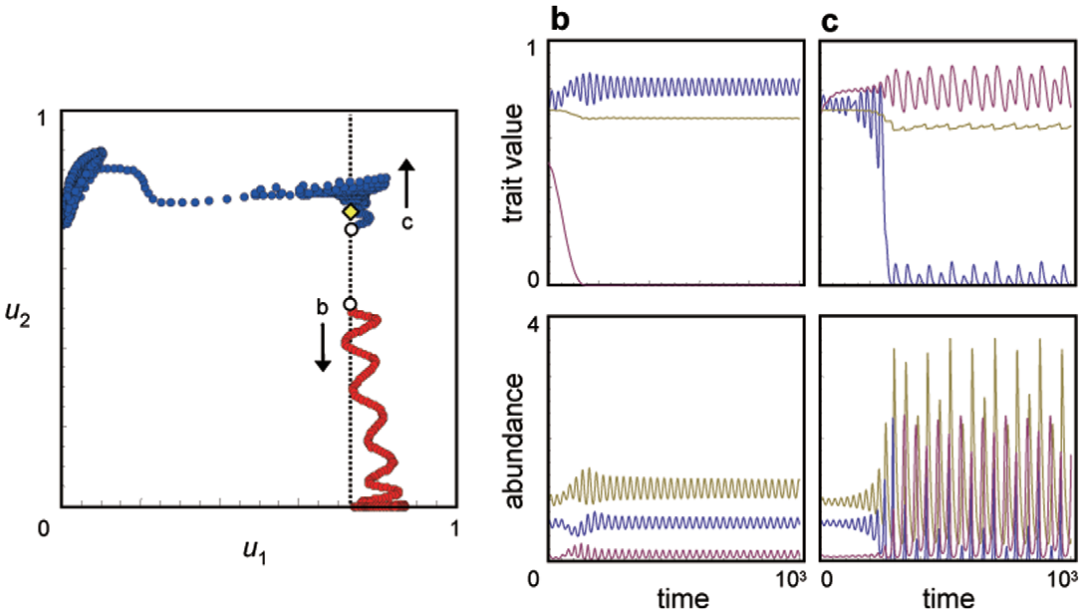
Three-species coevolutionary dynamics after the invasion of prey species 2 into an unstable one predator–one prey system. I assumed that the system without prey species 2 was at equilibrium. I also assumed *h*_1_ = *h*_2_ = 0.7. The other information is the same as in Fig. 3.

**Figure 5:**
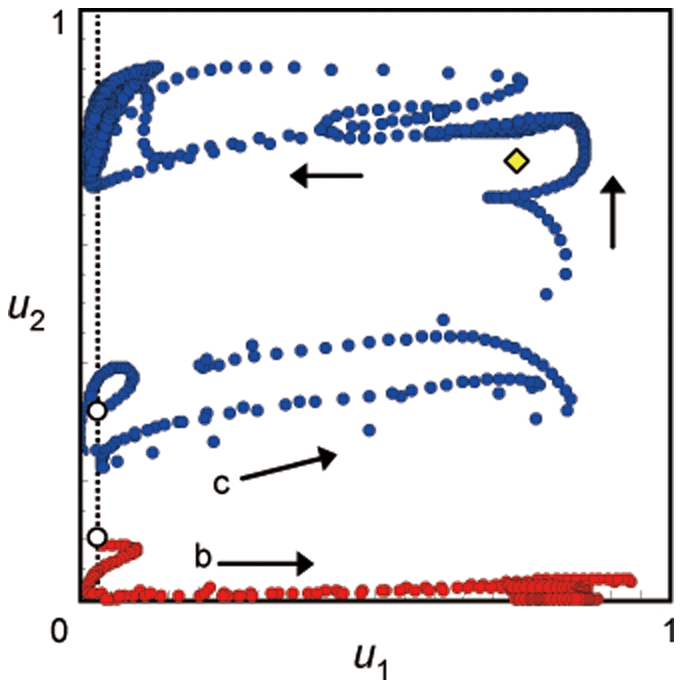
Example of three-species coevolutionary dynamics after the invasion of prey species 2 into an unstable one predator–one prey system showing population and trait value oscillations. The other information is the same as in Fig. 4.

Next, I consider the invasion of a second prey species into an original one predator–one prey system in which the population size and trait dynamics alternate between a steady state and an oscillatory state (Fig. 2e–h). The second prey with a small population size is introduced into the original one predator–one prey system at equilibrium (yellow diamond in Fig. 4a, S2a, b). If the initial trait value of the second prey is relatively lower than the equilibrium trait value of the resident prey (see the initial point of trajectory b in Fig. 4a), the defense trait of the second prey evolves to a lower value after the invasion, and the traits and populations of the three species exhibit cycles with smaller amplitudes than those in the original resident system (trajectory b in Fig. 4a; Fig. 4b). If the initial trait value of the second prey is close to the equilibrium trait value of the resident prey (see the initial point of trajectory c in Fig. 4a), the second prey evolves its defense to a higher level, and the resident prey evolves it defense to a much lower level, resulting in destabilization of the population dynamics (trajectory c in Fig. 4a; Fig. 4c). I also consider the invasion of the second prey into a resident system in which both population size and trait dynamics are in an oscillatory state. In such a system, population sizes and trait values can be far from the equilibrium, and the invasion of the second prey into the system can cause further destabilization to occur over a wide range of initial trait values of the second prey. The end result is like that shown in Fig. 4c. One example of this scenario is shown in Fig. 5. In this case, destabilization occurs even when the initial trait value of the second prey is very low (c in Fig. 5).

**Figure 6:**
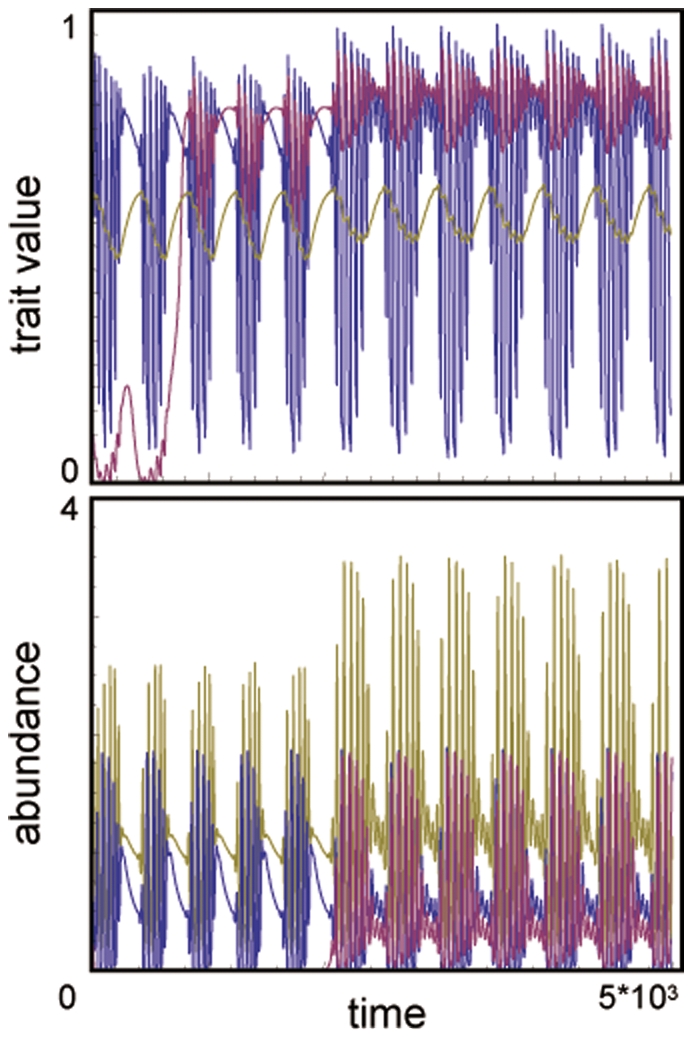
Example of three-species coevolutionary dynamics after the invasion of prey species 2 into an unstable one predator–one prey system showing population and trait value oscillations and under the assumption that *h*_1_ = *h*_2_ = 2.3. The other parameter values are the same as in Fig. 3.

Third, I consider the invasion of a second prey into a resident system in which the population size and trait dynamics are both in a perpetual oscillatory state (Fig. 2i–l). In this case, regardless of the invading prey's initial trait value, the second prey always evolves its defense to higher values and destabilizes the system (Fig. 6).

**Figure 7:**
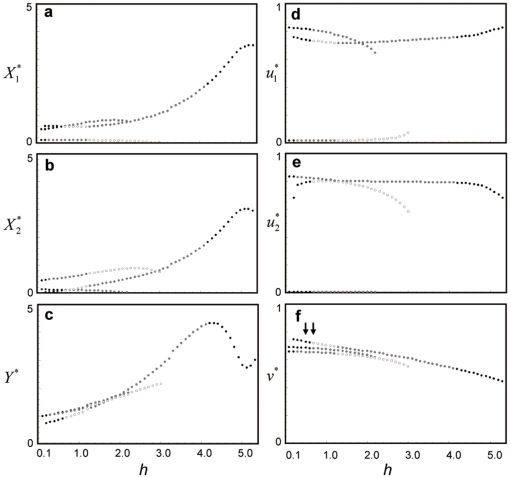
Equilibrium values in the three-species system in relation to handling time. The black, white, and gray points indicate locally stable and unstable equilibria and equilibrium that shows a limit cycle, respectively. The left and right arrows in the panel f indicate the parameter conditions which correspond to Fig. 3 and 4, respectively. Parameter values are the same as in Fig. 3.

**Figure 8:**
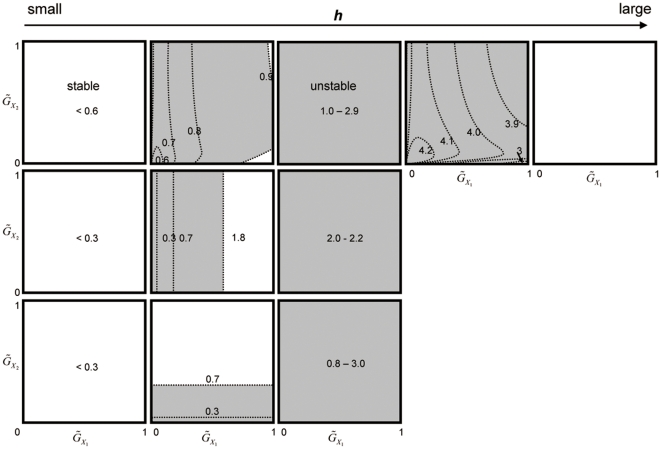
Parameter regions in which the equilibrium is stable or unstable. The two axes are 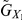 and 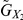. The white and shaded regions are the regions in which the equilibrium is stable and unstable, respectively. The numbers in the panels indicate the value of *h*, which becomes larger toward the right, as in Fig. 7. The upper, middle, and lower panels correspond to the stability region for the upper, middle, and lower equilibria shown in Fig. 7f.

The existence of multiple scenarios is due to the existence of multiple, locally stable equilibria or limit cycles in a coevolutionary system composed of three species (Fig. 7, 8, S3). The multiple trajectories in Fig. 3 and 4 correspond to the number of attractors in Fig. 7. In the case of Fig. 3, there are three possible outcomes, and in the case of Fig. 4, there are two possible outcomes. Note that the white circle is unstable node and the dynamics do not converge into this equilibrium.

**Figure 9:**
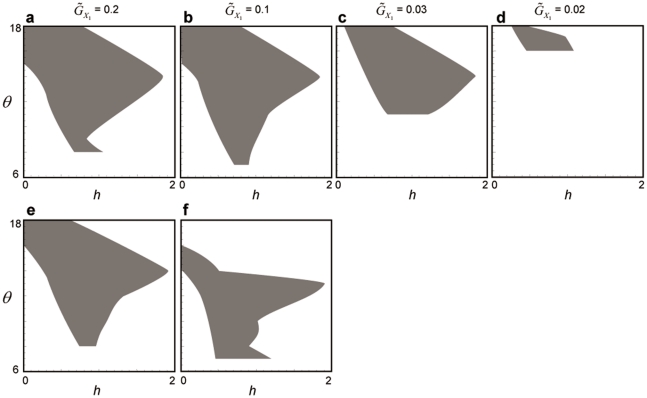
Parameter regions in which the prey species 2 takes over the coevolutionary dynamics. The initial trait values are (*u*_1_, *u*_2_, *v*) = (0.3, 0.1, 0.3). The shaded region indicates the parameter ranges in which a takeover of the dynamics occurs. (a–d) 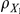 = 1.85. The value of 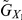 changes (shown above the panels) from left to right. (e) 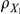 = 2. (f) 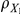 = 1.6. In both e and f, 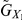 = 0.1. The other information is same as in Fig. S3.

Destabilization caused by evolution of the defense of the invading prey species and abandonment of its defense by the resident prey species (i.e., a ‘takeover of coevolution’) is likely to occur when the resident prey's defensive trait is less cost-constrained (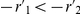) and the resident prey's speed of evolution is relatively faster (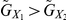) than the invading prey's (the resident prey is the adaptively superior species and the invading prey the adaptively inferior species) (Fig. 9).

In the opposite case, when the invading prey species is adaptively superior, the invading prey is likely to evolve its defense to lower values regardless of the initial trait value, resulting in destabilization of the population dynamics.

### Comparison with non-evolutionary system

I analyzed the equilibrium condition and the local stability of the equilibrium in the one predator-two prey system in which the all species do not evolve. The result shows that the coexistence equilibrium is always unique (Appendix S1). In addition, the invasion of alternative prey into one predator-one prey system can stabilize or destabilize the population dynamics, depending on the parameter values (Fig. S4). In contrast, the traits evolution can make multiple equilibria and can stabilize or destabilize the system, not depending on the parameter values but on the initial trait values. In addition, the evolution can change the stability of the system. As shown in Fig. 8, the large values of evolutionary speed of the preys tend to stabilize the system. In this figure, the predator's evolutionary speed is very small (0.005). In other words, the system is very close to non-evolutionary system when the values of evolutionary speed of the preys are close to zero. Thus, the evolution does not change the stability when the non-evolutionary system is stable. In contrast, fast evolution stabilizes the system when the non-evolutionary system is unstable. In other words, the evolution tends to stabilize the system.

## Discussion

Theory has shown that coevolutionary arms races driven by predator–prey interaction result in an equilibrium with higher trait values or in cycles [3]. In this study, I demonstrated that the invasion of an alternative prey species into such a coevolving predator and prey system greatly influences the coevolutionary dynamics. The effect of the invasion depends mainly on the initial trait value of the invading prey, the ease with which the prey can evolve (cost constraints and speed), and the stability of the resident system.

If the invading prey species has a relatively low defense ability when it invades, it is likely to evolve its defense to a lower level and the population dynamics are stabilized. In contrast, if the invading prey species has a defense ability close to that of the resident prey at the time of the invasion, the invading prey can evolve its defense to a higher level and the resident prey may suddenly cease to evolve its defense, resulting in destabilization of the population dynamics.

The mechanism of the first case is relatively simple. If the resident predator and prey have coevolved and their trait values are close to equilibrium, their trait values tend to be relatively high. As a result, the invading prey species with its lower defense level cannot lower predation pressure in the system. Thus, there is no selection pressure toward increased defense levels. In this case, if the resident prey is adaptively superior (its evolution is weakly constrained and fast) and the invading prey is adaptively inferior (its evolution is strongly constrained and slow), the predator can stably use the resident prey, which is well defended but maintains a large population, whenever the abundance of the less-defended invading prey population decreases.

In contrast, in the second case of an invading prey species with a relatively high defense level when it invades, both prey species evolve their defense, because by increasing their defense levels incrementally they can lower predation pressure in the system. If the invading prey is adaptively inferior, its defense level is evolutionarily maintained because its trait evolution has strong cost constraints and its adaptation speed is slow. An invading prey with a high defense level increases its abundance, which in turn decreases predation pressure on the superior prey, which then decreases its defense level. In this takeover of coevolution, however, the system's population dynamics are unstable because the less-defended resident prey, whose evolution is less constrained and faster, readily evolves its defense to some level such that the predator needs to use the well-defended invading prey very often.

I also found that a takeover of coevolution is likely to occur if a second prey invades when the resident system is unstable and shows population and trait value oscillations, because the predator's trait value in the original system oscillates and the difference between the invading prey's trait value and the predator's trait value will inevitably be sometimes small. Therefore, an unstable system is likely to be disturbed by an invading prey species not only because of its inherent ecological instability, but also because of the evolutionary instability.

These cases result in a great divergence of the traits of the two prey species, although the mechanism is different. Abrams [21] demonstrated in a theoretical study a similar divergence of traits in two prey to result from indirect evolutionary interaction mediated through a shared predator. He modeled several predator–prey systems in which two prey species could evolve their defense abilities but the predators could not evolve their offense abilities, and then compared the different evolutionary outcomes of each system. He found that great divergence was most often associated when two or more strategies for reducing predation risk were available or in the presence of two or more distinct types of predators with some form of trade-off in the preys' ability to avoid different predators. In other words, great divergence was not likely to occur when both prey species followed a single strategy for reducing predation by a single predator species. However, I showed here that great divergence can easily occur in such a system if the predator can also evolve. Such divergence may occur in three-spined sticklebacks [33], but the mechanism has not yet been demonstrated. Thus, the results presented here broaden the circumstances under which divergence by indirect evolutionary interaction through a shared predator can occur.

The present results show that whether coevolution in a predator–prey system is likely to be disturbed by an invading prey species can depend on the initial trait value of the invading prey. This suggests that the invasion of a prey species from a low-predation environment is not likely to disturb a resident interaction system in which the prey is exposed to high predation pressure. In other words, the effect of the invasion on the resident community may depend on the evolutionary histories of both the system from which the invading species comes and that which it invades. Recent theoretical studies have demonstrated that the evolutionary history of a community is an important determinant of it evolutionary fate [22], [34]. For example, in a mutualistic coevolutionary system, the invasion of an exploiter at an early stage of the mutualism's history can deflect the mutualists' coevolutionary trajectories toward different attractors and confer long-term stability against further exploitation [34]. In a prey–predator system, the timing of the invasion of a predator into an evolving prey community greatly influences the community's evolutionary fate [22]. These studies, however, did not consider the evolution of the third invading species. If that species also evolves, it cannot be excluded simply by a change in the evolutionary directions of the traits in the original interacting species. Instead, it may hijack the coevolutionary dynamics of the system.

## Supporting Information

Figure S1 Phase plot of the coevolutionary dynamics which correspond to Fig. 3.
(4.48 MB TIF)

Figure S2 Phase plot of the coevolutionary dynamics which correspond to Fig. 4.
(4.38 MB TIF)

Figure S3 Examples of nonequilibrium dynamics in relation to handling time for h1 = h2 = h. The other parameter values are the same as in Fig. 3. The initial abundance values are (X1, X2, Y) = (0.7, 0.1, 0.1). The initial value of u2 is 0.1, and the initial values of u1 and v, which were assumed to be same, are plotted as a white circle.
(5.51 MB TIF)

Figure S4 Parameter regions in which the equilibrium is stable or unstable in one predator-two prey system in which the all species do not evolve. The two axes are a2 and r2. The white and grey regions are the regions in which the equilibrium is stable and unstable, respectively. The black regions are the regions in which the three species cannot coexist. The asterisks in the panels indicate the values of a1 and r1 used in the stability analysis of three-species system. The bars in the upper side of each panel indicate the parameter space where the equilibrium is stable or unstable in the absence of alternative prey (X2 = 0). The colors correspond to those in lower panels. The focal parameter is a1. The local stability condition in one predator-one prey system is known: a1K1>(bg1+dh1)/ h1 (bg1-dh1). This means that r1 does not influence the stability. Thus I only focus on the dependence of a1 on the stability. Note that r influence the stability in the three-species system. The asterisks in the bars indicate the value of a1 used in the stability analysis of three-species system. (a) h1 = h2 = h = 0.1. (b) h = 0.3. (c) h = 0.6. (d) h = 2. Other parameter values are r1 = 0.9, K1 = K2 = 4, b = 0.5, g1 = g2 = 1, a1 = 0.6, and d = 0.1.
(4.95 MB TIF)

Appendix S1 Analysis of the one predator-two prey system in the absence of evolution.
(0.05 MB DOC)
