# Supplementary figures and images for: Coevolution in a One Predator–Two Prey System
Source: PLoS One. 2010 Nov 9;5(11):e13887. doi: 10.1371/journal.pone.0013887 (PMC2976687; doi:10.1371/journal.pone.0013887)

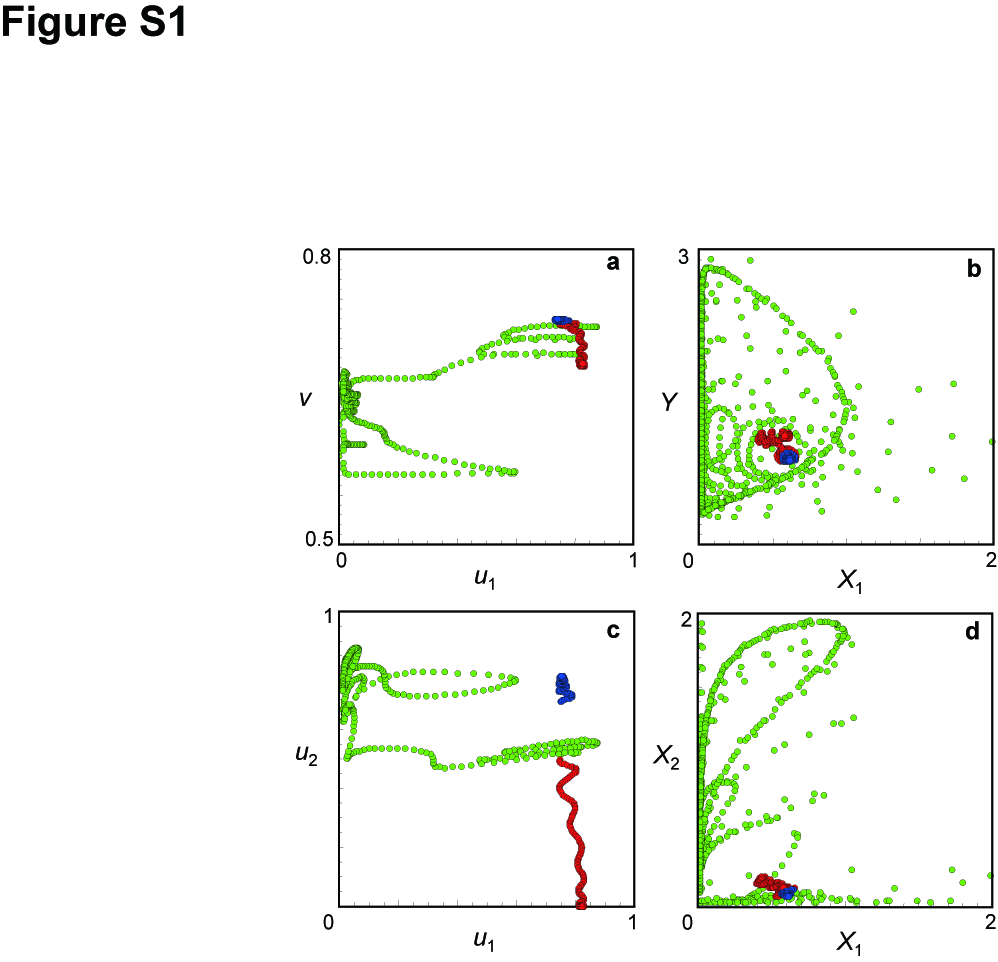

Supplement: Figure S1 — Phase plot of the coevolutionary dynamics which correspond to Fig. 3. (4.48 MB TIF) [file pone.0013887.s001.tif]

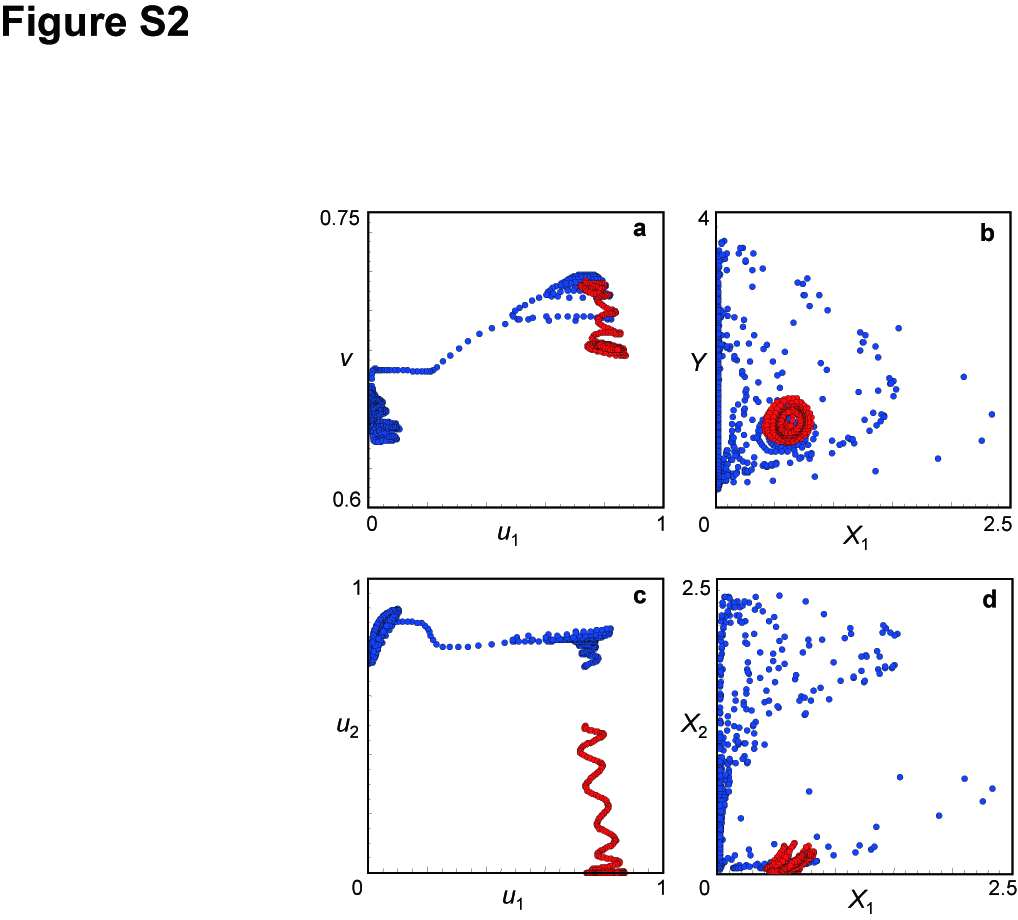

Supplement: Figure S2 — Phase plot of the coevolutionary dynamics which correspond to Fig. 4. (4.38 MB TIF) [file pone.0013887.s002.tif]

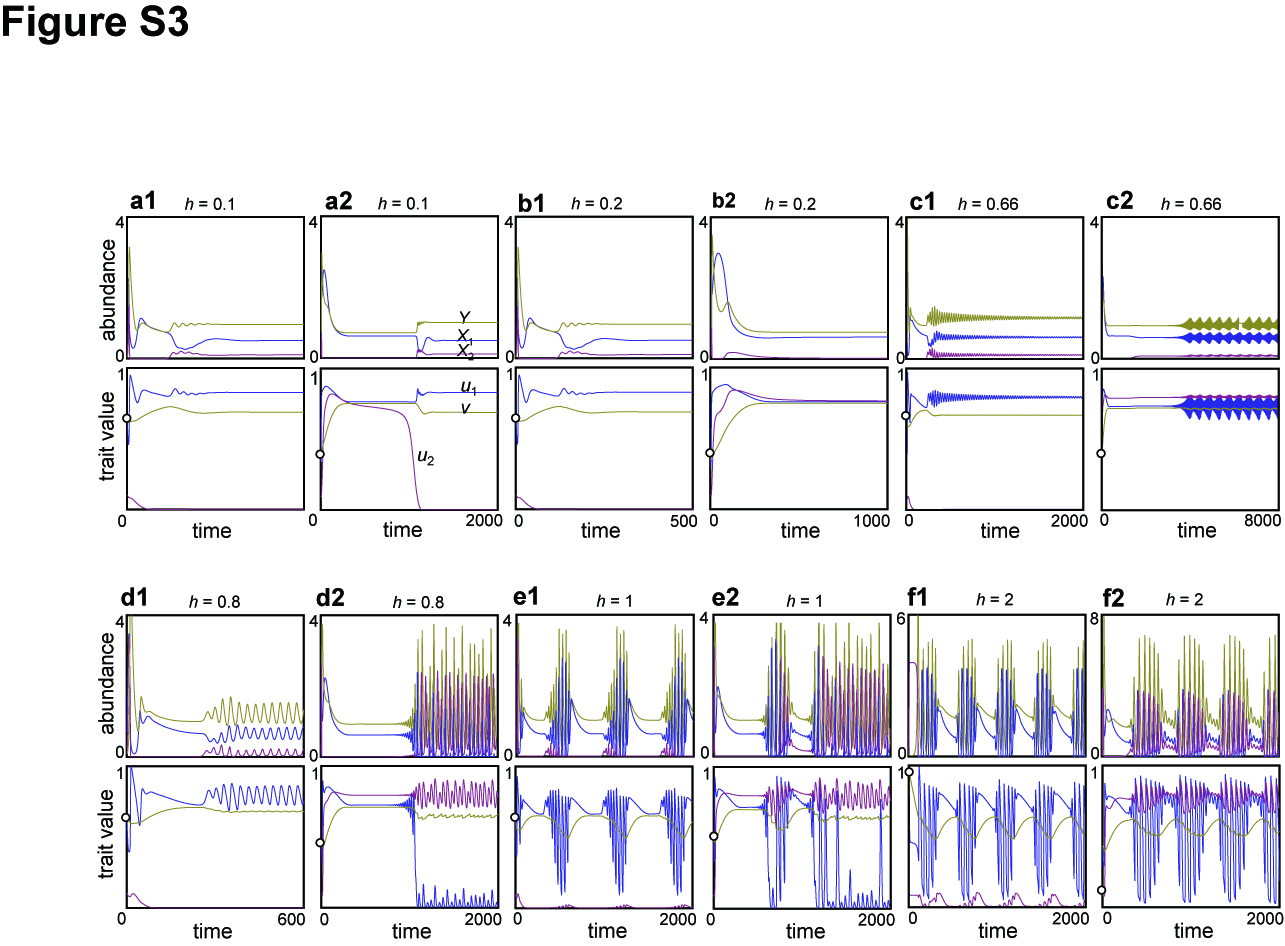

Supplement: Figure S3 — Examples of nonequilibrium dynamics in relation to handling time for h1 = h2 = h. The other parameter values are the same as in Fig. 3. The initial abundance values are (X1, X2, Y) = (0.7, 0.1, 0.1). The initial value of u2 is 0.1, and the initial values of u1 and v, which were assumed to be same, are plotted as a white circle. (5.51 MB TIF) [file pone.0013887.s003.tif]

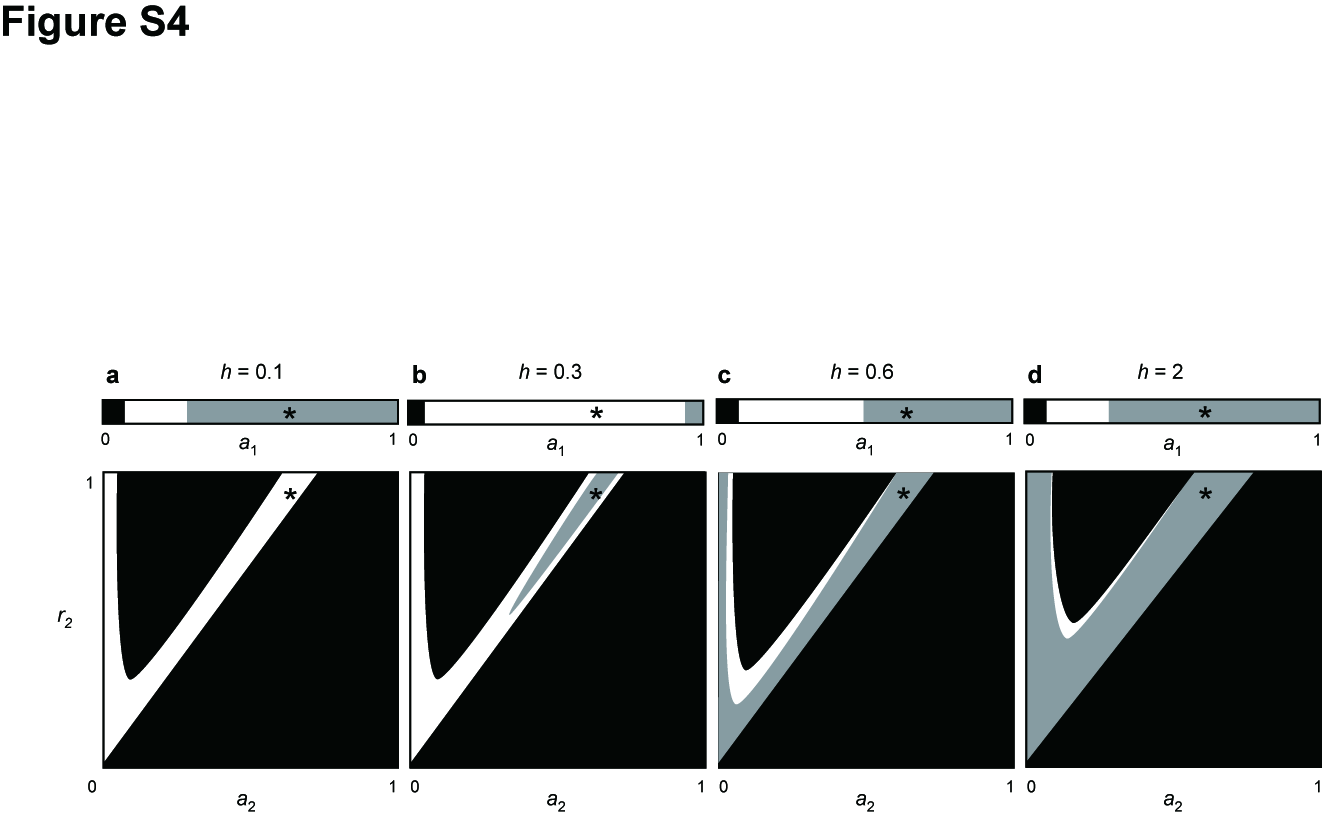

Supplement: Figure S4 — Parameter regions in which the equilibrium is stable or unstable in one predator-two prey system in which the all species do not evolve. The two axes are a2 and r2. The white and grey regions are the regions in which the equilibrium is stable and unstable, respectively. The black regions are the regions in which the three species cannot coexist. The asterisks in the panels indicate the values of a1 and r1 used in the stability analysis of three-species system. The bars in the upper side of each panel indicate the parameter space where the equilibrium is stable or unstable in the absence of alternative prey (X2 = 0). The colors correspond to those in lower panels. The focal parameter is a1. The local stability condition in one predator-one prey system is known: a1K1>(bg1+dh1)/ h1 (bg1-dh1). This means that r1 does not influence the stability. Thus I only focus on the dependence of a1 on the stability. Note that r influence the stability in the three-species system. The asterisks in the bars indicate the value of a1 used in the stability analysis of three-species system. (a) h1 = h2 = h = 0.1. (b) h = 0.3. (c) h = 0.6. (d) h = 2. Other parameter values are r1 = 0.9, K1 = K2 = 4, b = 0.5, g1 = g2 = 1, a1 = 0.6, and d = 0.1. (4.95 MB TIF) [file pone.0013887.s004.tif]
