# Supplementary material for: Coevolution in a One Predator–Two Prey System
Source: PLoS One. 2010 Nov 9;5(11):e13887. doi: 10.1371/journal.pone.0013887 (PMC2976687; doi:10.1371/journal.pone.0013887)
Supplement: Appendix S1 — Analysis of the one predator-two prey system in the absence of evolution. (0.05 MB DOC) [file pone.0013887.s005.doc]

**Appendix S: Analysis of the one predator-two prey system in the absence of evolution**

I study the equilibrium condition of the system described by Eqs. (1). I assume that *K*_1_ = *K*_2_ = *K* and *h*_1_ *=h*_2_ = *h* as assumed in the text. Then I can obtain unique coexistence equilibrium,

$X_{1}^{*}=\frac{Ka_{2}\left(g-dh\right)\left(a_{2}r_{1}-a_{1}r_{2}\right)+a_{1}r_{2}d}{\left(g-dh\right)\left(a_{2}^{2}r_{1}+a_{1}^{2}r_{2}\right)},$ (A1a)

$X_{2}^{*}=\frac{Ka_{1}\left(g-dh\right)\left(a_{1}r_{2}-a_{2}r_{1}\right)+a_{2}r_{1}d}{\left(g-dh\right)\left(a_{2}^{2}r_{1}+a_{1}^{2}r_{2}\right)},$ (A1b)

$Y^{*}=\frac{gr_{1}r_{2}\left\{K\left(a_{1}+a_{2}\right)\left(g-dh\right)-d\right\}}{K\left(g-dh\right)^{2}\left(a_{2}^{2}r_{1}+a_{1}^{2}r_{2}\right)}$. (A1c)

I find that the conditions, $X_{1}^{*}$ > 0,$X_{2}^{*}$ > 0, and *Y^*^* > 0, are

$K\left(\frac{g}{d}-h\right)\left(1-\frac{a_{2}r_{1}}{a_{1}r_{2}}\right)<\frac{1}{a_{2}},$ (A2a)

$K\left(\frac{g}{d}-h\right)\left(1-\frac{a_{1}r_{2}}{a_{2}r_{1}}\right)<\frac{1}{a_{1}},$ (A2b)

$K\left(\frac{g}{d}-h\right)>\frac{1}{a_{1}+a_{2}},$ (A2c)

respectively. First I find that (A2c) necessarily requires the condition *g* > *dh*. When *a*_1_/*a*_2_ > *r*_1_/*r*_2_, the condition of coexistence equilibrium is

$\frac{1}{a_{1}+a_{2}}<K\left(\frac{g}{d}-h\right)<\frac{a_{1}r_{2}}{\left(a_{1}r_{2}-a_{2}r_{1}\right)a_{2}}$. (A3)

When *a*_1_/*a*_2_ < *r*_1_/*r*_2_, the condition of coexistence equilibrium is

$\frac{1}{a_{1}+a_{2}}<K\left(\frac{g}{d}-h\right)$ (A4a)

and

$\frac{a_{2}r_{1}}{\left(a_{2}r_{1}-a_{1}r_{2}\right)a_{1}}<K\left(\frac{g}{d}-h\right)$. (A4b)

Next, I study the local stability of the equilibrium by linearizing the dynamics near the nontrivial equilibrium. I can judge the local stability by examining the sign of the dominant eigenvalue of their Jacobian matrix. The numerical analysis is shown in Fig. S8.
